# Optimization of a CRISPR/Cas9-mediated Knock-in Strategy at the Porcine Rosa26 Locus in Porcine Foetal Fibroblasts

**DOI:** 10.1038/s41598-017-02785-y

**Published:** 2017-06-08

**Authors:** Zicong Xie, Daxin Pang, Kankan Wang, Mengjing Li, Nannan Guo, Hongming Yuan, Jianing Li, Xiaodong Zou, Huping Jiao, Hongsheng Ouyang, Zhanjun Li, Xiaochun Tang

**Affiliations:** Jilin Provincial Key Laboratory of Animal Embryo Engineering, College of Animal Sciences, Jilin University, Changchun, Jilin Province People’s Republic of China

## Abstract

Genetically modified pigs have important roles in agriculture and biomedicine. However, genome-specific knock-in techniques in pigs are still in their infancy and optimal strategies have not been extensively investigated. In this study, we performed electroporation to introduce a targeting donor vector (a non-linearized vector that did not contain a promoter or selectable marker) into Porcine Foetal Fibroblasts (PFFs) along with a CRISPR/Cas9 vector. After optimization, the efficiency of the EGFP site-specific knock-in could reach up to 29.6% at the pRosa26 locus in PFFs. Next, we used the EGFP reporter PFFs to address two key conditions in the process of achieving transgenic pigs, the limiting dilution method and the strategy to evaluate the safety and feasibility of the knock-in locus. This study demonstrates that we establish an efficient procedures for the exogenous gene knock-in technique and creates a platform to efficiently generate promoter-less and selectable marker-free transgenic PFFs through the CRISPR/Cas9 system. This study should contribute to the generation of promoter-less and selectable marker-free transgenic pigs and it may provide insights into sophisticated site-specific genome engineering techniques for additional species.

## Introduction

Genetically modified pigs have provided numerous advantages to agriculture and are also widely used to study human diseases^1^. Traditional homologous recombination (HR) can be used to generate transgenic pigs, but its low efficiency and laborious and time consuming properties remain problematic. Thus, the generation of transgenic pigs would benefit from a more ideal gene editing tool, a “safe harbour” locus in the pig genome and efficient gene targeting strategies.

The Rosa26 locus is a “safe harbour” and is conserved in multiple species, including mice^2^, human^3^, rats^4^, pigs^5^, sheep^6^ and rabbits^7^. The sequence of the pig ROSA26 (pROSA26) locus has been completely characterized, and the pRosa26 promoter has been identified^8^. This porcine endogenous promoter is suitable for driving exogenous gene expression in a high and stable manner by avoiding DNA methylation.

With the recent development of site-specific nucleases, including zinc finger nucleases (ZFNs), transcription activator-like effector nucleases (TALENs) and clustered regularly interspersed short palindromic repeat (CRISPR)/CRISPR-associated protein 9 (Cas9), it has become possible to generate transgenic animals with a high efficiency. The CRISPR/Cas9 system is an ideal gene editing tool, and its high efficiency, rapid assembly, low cost and ease of use have enabled its wide use in various species^9–12^. However, even with CRISPR/Cas9, the knock-in efficiency (particularly for pigs) remains low, and optimal strategies have not been extensively investigated. Here, we improved upon several knock-in related conditions, including the transfection efficiency and optimal transfection dosage of CRISPR/Cas9 and a suitable homology arm length for efficient homologous recombination (HR). Our results indicate that CRISPR/Cas9-mediated genome engineering can be successfully adopted to efficiently generate promoter-less and selectable marker-free transgenic PFFs. The purpose of this study was to serve utilization of somatic cell nuclear transfer (SCNT) to generate transgenic pigs^11, 13^. So, the EGFP reporter PFFs were further used to evaluate two key factors in the process of achieving transgenic pigs the limiting dilution method and a strategy to evaluate the safety and feasibility of the knock-in locus. In summary, this study provides a simple and efficient approach for a promoter-less and selectable marker-free site-specific knock-in strategy and contributes towards the development of transgenic pigs.

## Results

### Establishing an efficient and versatile electroporation system

**Figure 1:**
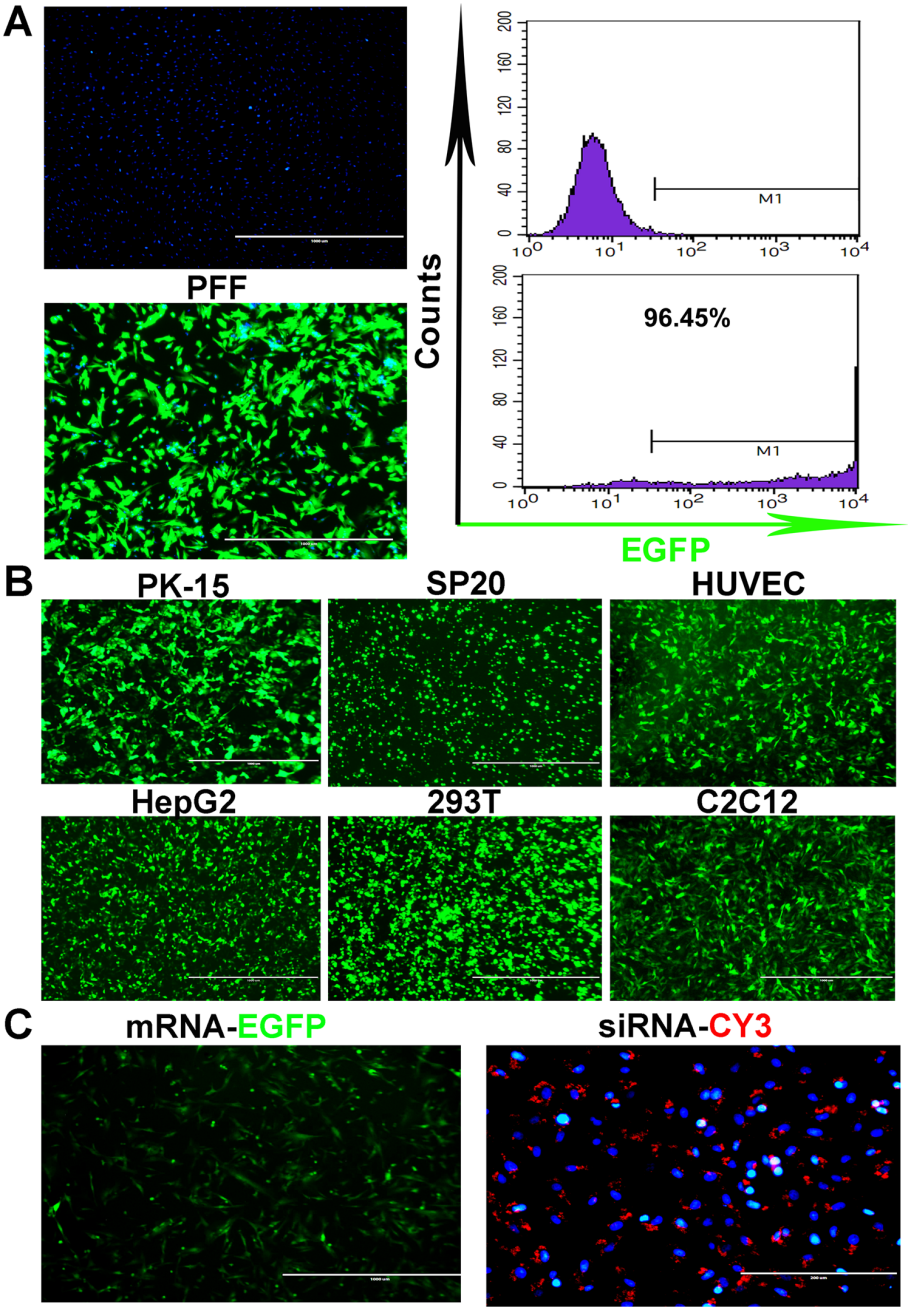
An efficient and versatile electroporation system. (**A**) Transient transfection of the pEGFP-N1 vectors into the PFFs. The transfection efficiency was analysed with fluorescence microscopy and FACS at 24 hours post-transfection. (**B**) Transient transfection of the pEGFP-N1 vector into the indicated cell lines. EGFP fluorescence was analysed via fluorescence microscopy. (**C**) Transfection of the EGFP mRNA and CY3-labelled siRNA into PFFs. The fluorescence was analysed via fluorescence microscopy.

The transfection efficiency of the PFFs was a barrier against generating transgenic pigs by SCNT. Our group transiently transfected pEGFP-N1 plasmids into PFFs in a previous study, approximately 90% of the surviving cells displayed green fluorescence^14^. After further optimization, the electroporation efficiency of the PFFs reached approximately 96.45% (Fig. 1A). Additionally, we confirmed that this electroporation system had high transfection efficiency in various other cell lines (Fig. 1B and Supplementary Fig. S1), and the electroporation parameters for those cell lines are listed in Table 1. Interestingly, the EGFP mRNA (10 μg) and the Cy3-siRNA (Cy3 labelled siRNA, 100 nM) were also successfully and efficiently introduced into the PFFs by the electroporation system, and the EGFP and the Cy3-positive cells were observed by fluorescence microscopy at 24 hours post-transfection and 3 hours post-transfection, respectively (Fig. 1C).

**Table 1:** Parameters of various cell lines for electroporation (BTX-ECM2001).

Cell lines	Parameters For Electroporation(BTX)		
	Set Voltage(V)	Pulse Length (ms)	Number of Pulses
PFF	340	1	3
PK-15	300	1	3
Vero	280	1	3
HUVEC	220	1	3
HepG2	240	1	3
293T	220	1	3
C2C12	250	1	3
SP20	220	1	3

### Optimization of the targeting/cutting efficiency at the PFF pRosa26 locus

**Figure 2:**
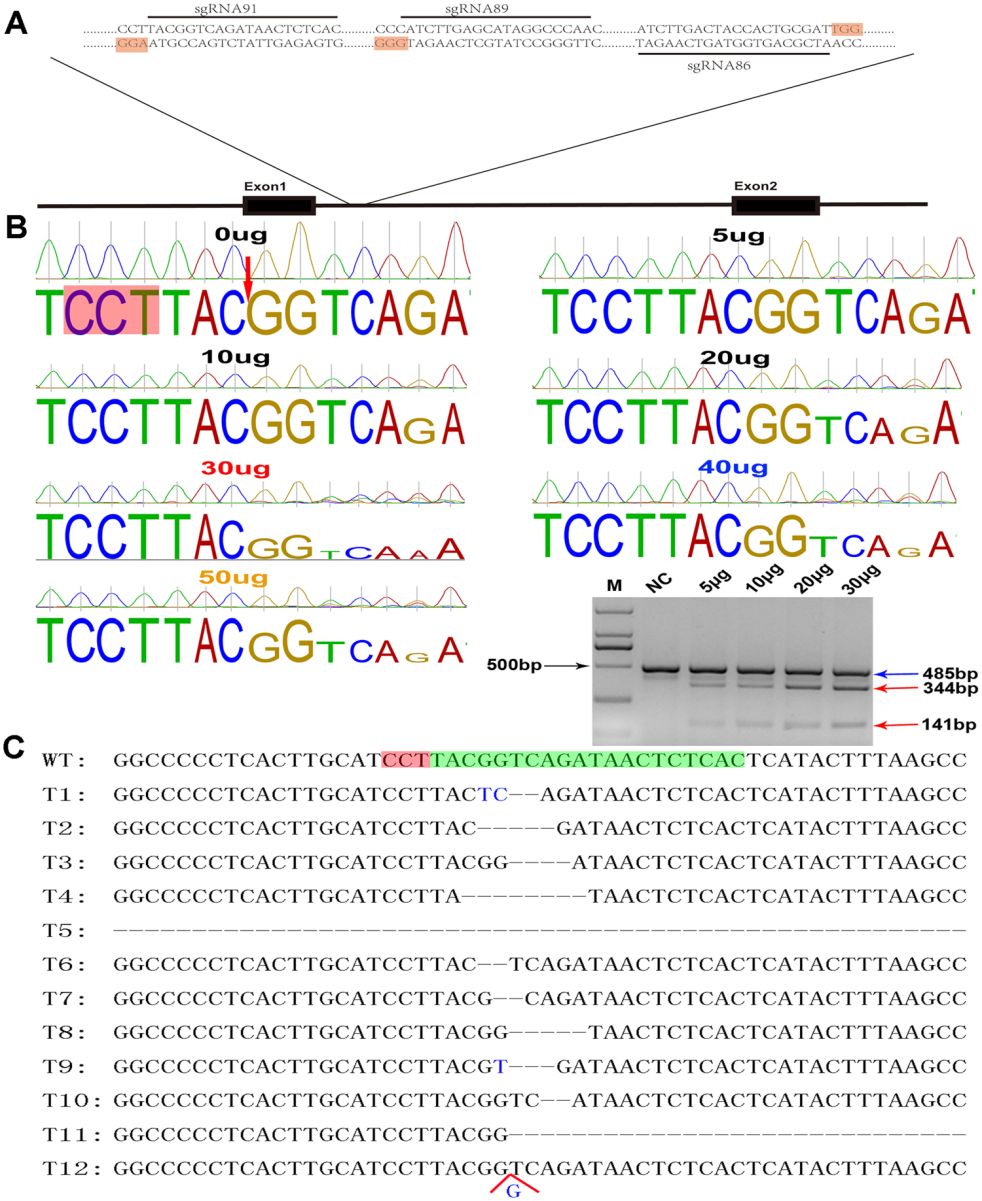
Optimization of the mutation/cutting efficiency at the pROSA26 locus in PFFs. (**A**) Scheme for site-specific targeting/cutting of the pROSA26 locus on chromosome 13 by CRISPR/Cas9. (**B**) Chromatograms of the PCR amplicons for each CRISPR/Cas9 dose group (0 μg, 5 μg, 10 μg, 20 μg, 30 μg, 40 μg and 50 μg). The red arrow indicates the cleavage site, and the PAM is underlined in red. The mutation efficiency for each CRISPR/Cas9 dose (5 µg, 10 µg, 20 μg and 30 µg) was determined by using the T7E1 cleavage assay. M: 100 bp DNA ladder. NC: Negative control, transfection dose of CRISPR/Cas9 was 0 µg. The blue arrows indicated wild type bands and the red arrows indicated cleaved amplicons. (**C**) The TA cloning and sanger sequences were analysed for indels. The wild-type sequence is located on the first line (WT), and the mutated sequences from the TA cloning are arranged below (T1~T12). The target site is indicated in green; the PAM is indicated in red.

The endogenous pRosa26 promoter was utilized to express the exogenous gene as shown in the Fig. 2A. According to a previous report on the pRosa26 gene^5^, three sgRNAs were designed to target intron 1 of the pRosa26 gene. To obtain an efficient sgRNA, three sgRNA-recombined PX330 plasmids were introduced into PFFs by electroporation. After 3 days in culture, the targeting/cutting efficiencies of the three sgRNAs were measured via T7 endonuclease I (T7EI) assays and confirmed by sequencing; the results showed that sgRNA91 achieved a higher targeting/cutting efficiency than sgRNA86 and sgRNA89 (Supplementary Fig. S2). To our knowledge, different transfection systems/methods have different requirements regarding the amounts and concentrations of the CRISPR/Cas9 vectors, and suitable transfection dosages of the CRISPR/Cas9 vectors should achieve higher cutting/targeting efficiencies with less cytotoxicity. Therefore, different doses of the sgRNA91/Cas9 vectors were introduced into the PFFs, and the results of the sequencing and T7EI digestion assays showed that the 30 μg group achieved a higher targeting/cutting efficiency than that of the other groups (Fig. 2B). Additionally, the TA cloning results confirmed that the mutation efficiency of sgRNA91/Cas9 was 34.3% (Fig. 2C and Supplementary Table S1). Although the 40 μg and 50 μg groups also achieved high targeting/cutting efficiencies (Fig. 2B), they produced higher cytotoxicities (Supplementary Fig. S3). Several groups have efficiently obtained genetically modified mice and pigs by injecting the DNA or mRNA of site-specific nucleases into zygotes to achieve targeted mutations^15, 16^. Because of the low cost, ease and rapidity of the parthenogenote injection, we planned to evaluate the targeting/cutting efficiencies of three sgRNAs (sgRNA91, sgRNA86 and sgRNA89) and reconfirm the higher targeting/cutting efficiency of sgRNA91 for the following experiments. SgRNA-91 (20 ng/µL) and the Cas9 mRNA (40 ng/µL) were injected into the cytoplasms of parthenogenetic embryos; the results showed that the mutation efficiency of the parthenogenetic embryos was only 14. 8% (8/54), and the development rate of the parthenogenetic embryos was only 8.3% (34/410) (Supplementary Fig. S4). The development rate of the parthenogenetic embryos was lower than that of the control group, which was 11% (44/396).

### Optimization of the site-specific EGFP insertion at the PFF pRosa26 locus

**Figure 3:**
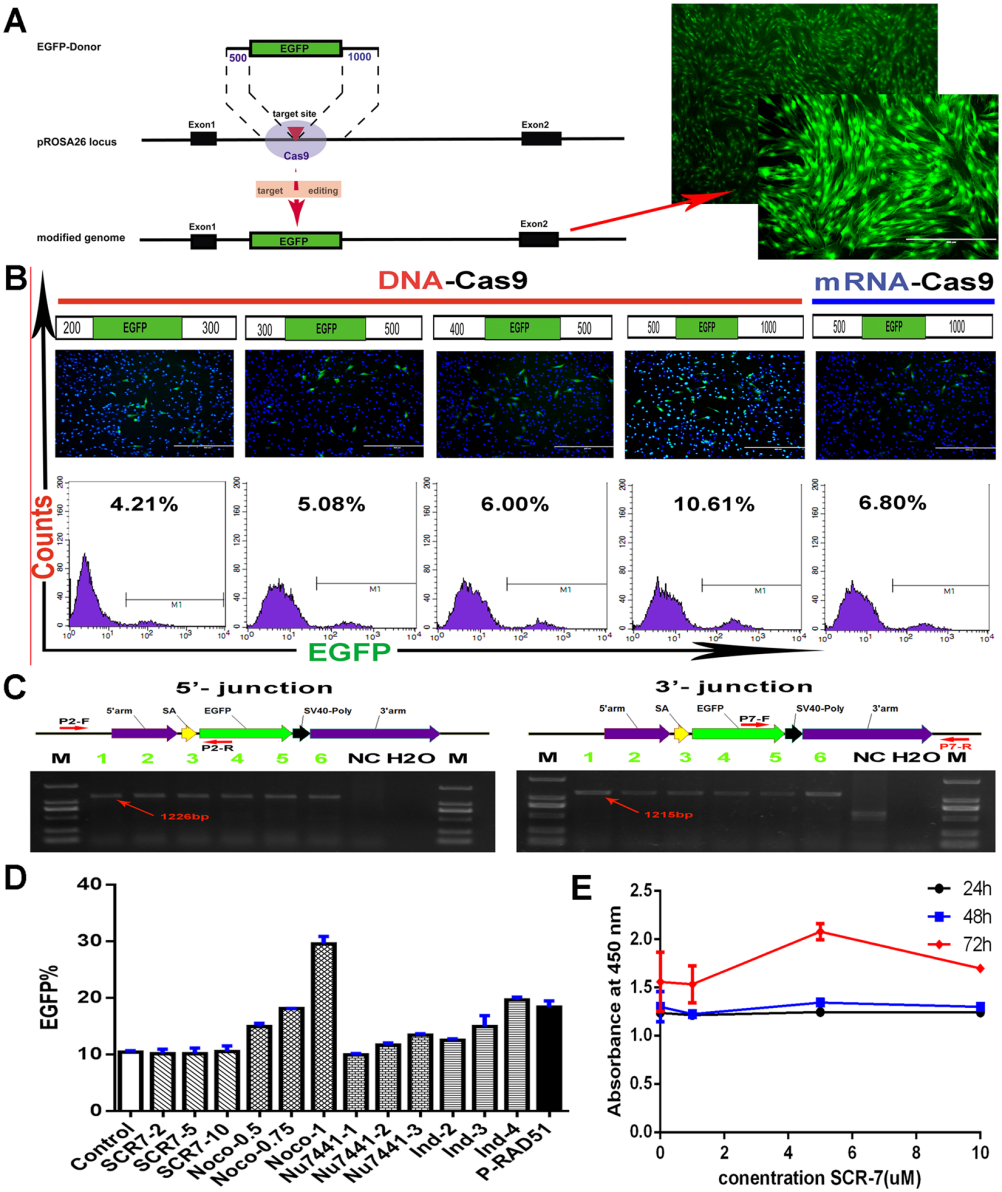
Optimization of the knock-in efficiency by EGFP site-specific integration at the pROSA26 locus. (**A**) Scheme for EGFP site-specific knock-in at the pROSA26 locus. The EGFP-KI- positive PFF cell clone indicated with a red arrow. (**B**) The effect of the homology arm lengths on the EGFP site-specific knock-in in PFFs. EGFP fluorescence was analysed via fluorescence microscopy and FACS, at three days post-transfection. Detailed statistical analyses were showed in Fig. S6. (Remarks: the control group that only the circular EGFP targeting donor vector introduced into the PFFs and without the CRISPR/Cas9 vector). (**C**) The result of the genomic PCR analysis confirmed the knock-in events at the pROSA26 locus. P2 primers amplified the 5′- junction and P7 primers amplified the 3′- junction; the sequences of these primers are listed in Table S3. Lanes 1–6 represent the EGFP knock-in-positive cell clones. NC: Negative control; M: D2000. (**D**) The effect of different drugs and concentrations on EGFP site-specific knock-in in PFFs. (Column chart, n = 3 independent experiments). Control, DMSO control; SCR7-2, SCR7(2 μM); SCR7-5, SCR7 (5 μM); SCR7-10, SCR7 (10 μM); Noco-0.5, Nocodazole (0.5 μg/ml); Noco-0.75, Nocodazole (0.75 μg/ml); Noco-1, Nocodazole (1 μg/ml);Nu7441-1, Nu7441 (1 μM); Nu7441-2, Nu7441 (2 μM); Nu7441-3, Nu7441(3 μM); Ind-2, indirubin-3′-monoxime (2 μg/ml); Ind-3, indirubin-3′-monoxime (3 μg/ml); Ind-4, indirubin-3′-monoxime (4 μg/ml); P-RAD51, pig RAD51 over expression plasmid. (**E**) The effect of different concentrations of SCR7 on PFFs proliferation. (Line chart. n = 3. Graphs show the mean ± S.E.M.).

On the basis of our preceding work mentioned above, to achieve a one-step transgenic strategy, a non-linearized targeting donor vector (PUC57-pRosa26-EGFP) that did not contain a promoter or a selectable marker was used in this study (Supplementary Fig. S5). The EGFP knock-in strategy is shown in Fig. 3A. First, the homology arm length was evaluated for efficient homologous recombination (HR) in PFFs. The donor vectors that contained different homologous arm lengths (0.5 kb, 0.8 kb, 0.9 kb and 1.5 kb) were constructed and introduced into PFFs along with the sgRNA91/Cas9 vector. On the basis of the control group (only non-linearized targeting donor vectors were introduced into the PFFs and without the CRISPR/Cas9 vectors), the fluorescence microscopy and FACS results showed that all four donors produced site-specific EGFP insertion into the PFF pRosa26, and the 1.5 kb group (10.4%) had a higher knock-in efficiency than did the other groups (3.1%, 4.3% and 5.5%) (Fig. 3B, Supplementary Fig. S6). The site-specifically inserted EGFP was confirmed by PCR with specific primers (Fig. 3C), and the sequencing results are shown in Supplementary Fig. S6. As indicated above, our electroporation system was also suitable for mRNA transfections. Therefore, the *in vitro* transcribed Cas9 mRNA and sgRNA91 were introduced into the PFFs with the EGFP donor vector by electroporation. Three days later, the EGFP-positive cells were observed by fluorescence microscopy and analysed by FACS. Interestingly, the results confirmed that the EGFP site-specific knock-in was also achieved in PFFs by electroporation of the Cas9 mRNA and sgRNA (6.5%) (Fig. 3B, Supplementary Fig. S6).

The SCR7 ligase IV inhibitor reportedly increases the efficiency of HDR-mediated genome editing in several mammalian cell lines^2, 17^. Given this observation, we combined multiple SCR7 doses with the electroporation buffer solution (the electroporation system) and cell culture medium (the cell culture system). First, we combined different doses of SCR7 (1 μM, 2 μM, 5 μM and 10 μM) and the pEGFP-N1 plasmids (30 μg) into the electroporation system, and the fluorescence microscopy analysis results at 24 hours post-transfection showed that the 2 μM group, compared with other group, was introduced into the PFFs with a high efficiency and no apparent cytotoxicity (Supplementary Fig. S7). Our results demonstrated that no noticeable enhancement to the HDR efficiency. However, the CCK-8 analysis confirmed that SCR7 (5 μM) could significantly promoted cell proliferation in the cell culture system (Fig. 3E and Supplementary Fig. S8). On the basis of these results, the 2 μM SCR7 parameter from the transfection system and the 5 μM SCR7 parameter from the culture system, those were selected to explore the SCR7 effectiveness with the EGFP site-specific knock-in events. However, there was no noticeable enhancement of the HDR efficiency in our system.

We next wished to directly compare the knock-in efficiency stimulated by SCR7 to that of other drugs recently reported, including the cyclin-dependent kinase (CDK) inhibitor indirubin-3′-monoxime^18^, DNA-PKcs inhibitors NU7441^19^ and microtubule polymerization inhibitor Nocodazole^20^. After transfection, PFFs were incubated for 6 hours in drug-free medium, and then, replaced the drug-free medium with different concentrations of drug medium to allow the knock-in of the EGFP-KI donor. 72 hours later, the EGFP-positive cells were observed by fluorescence microscopy and analysed by FACS. Our result showed that synchronization with Nocodazole (1 µg/ml) resulted in a 2.8-fold increase (29.6%) (Fig. 3D), indirubin-3′-monoxime (4 µg/ml) treatment had a 1.9-fold increase (19.7%) (Fig. 3D). However, the SCR7 and NU7441 treatment did not show increase of knock-in efficiency compared to control (Fig. 3D).

Additionally, previous study showed that RAD51 recombinase was a key player for HR and the repair of DNA DSBs^21^ and the cDNA sequence of pig RAD51 gene was successfully isolated by PCR-based methods^22^. In order to investigate RAD51 effects on the indicated pROSA26 site insertion, we constructed pig RAD51-over expression plasmid (Supplementary Fig. S9), and then introduced the plasmid with sgRNA91/Cas9 and the EGFP donor vector into PFFs, 72 hours later, the EGFP-positive cells were observed by fluorescence microscopy and analysed by FACS. The results showed that the RAD51-over expression plasmid could result in a 1.8-fold increase (18.4%) in the indicated PFFs system (Fig. 3D).

### Off-target analysis

**Figure 4:**
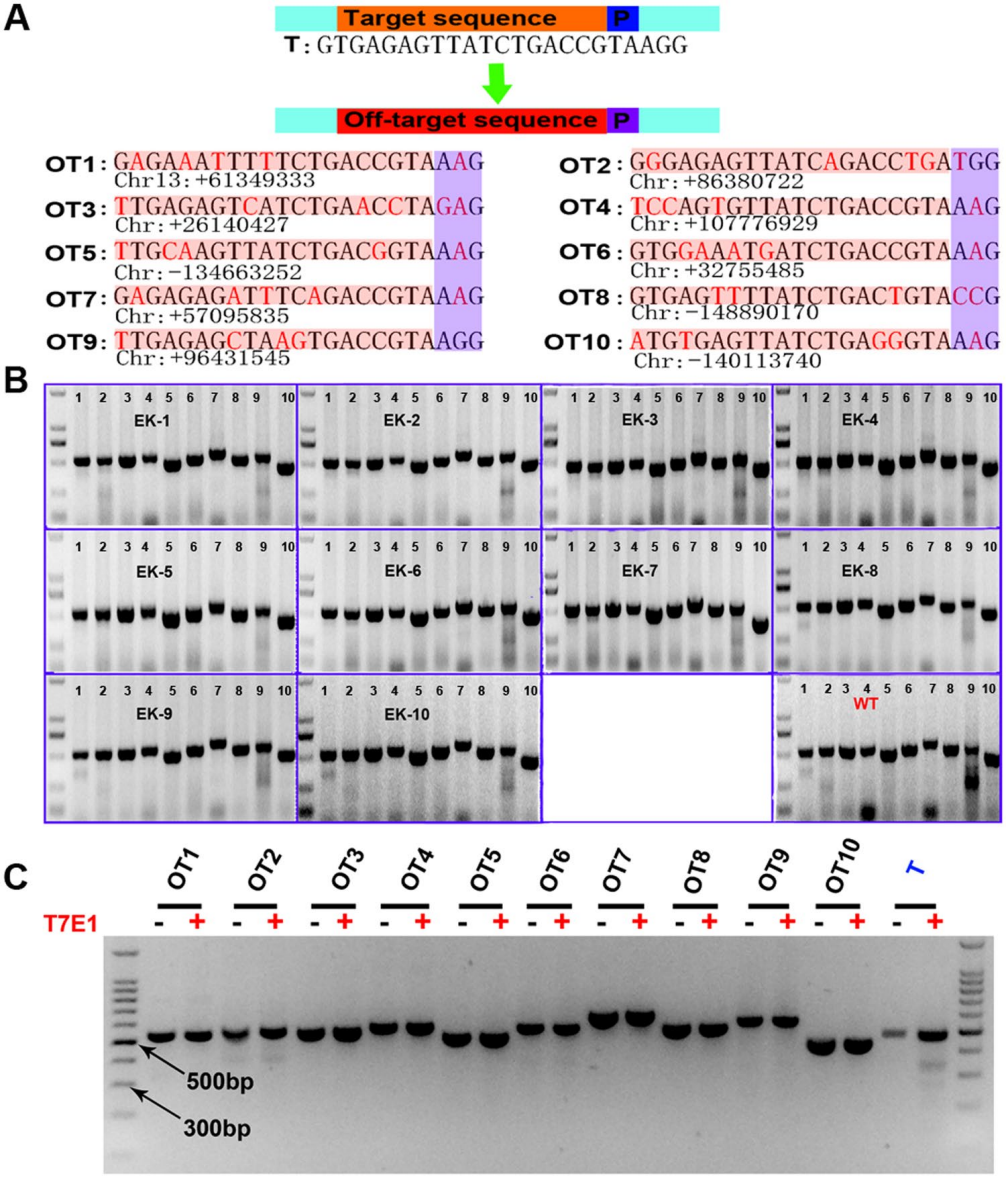
Off-target analysis. (**A**) The target sequence and ten of the predicted off-target sites for sgRNA91/Cas9. The PAM sequence is labeled in blue. (**B**) A total of ten potential off target loci (Lanes 1–10) were selected to examine off target effects in EGFP site-specific knock-in cell clone. In order to further confirm the specificity of sgRNA91/Cas9, ten EGFP site-specific knock-in cell clones (EK1-EK10) were selected randomly were evaluated by PCR and sequencing. WT: Wild-type. (**C**) T7E1 assays for the ten potential off-target sites. OT1-OT10 represents 10 potential off-target sites, and T represents target site.

Off-target effects by CRISPR/Cas9 have been reported, owing to the system’s ability to tolerate the sequence mismatches^15, 23^. Off-target effects may affect the development of the blastocyst and the generation of transgenic pigs. Therefore, 10 potential off-target sites were selected according to the online CRISPR design (Fig. 4A), and 10 random EGFP-positive cell clones were evaluated (Fig. 4B). The DNA sequencing and T7E1 assay showed that no off-target mutations were detectable in any of the potential off-target sites or these EGFP-positive cell clones (Fig. 4B,C and Supplementary Fig. S10).

### Application of the EGFP knock-in reporter PFFs

**Figure 5:**
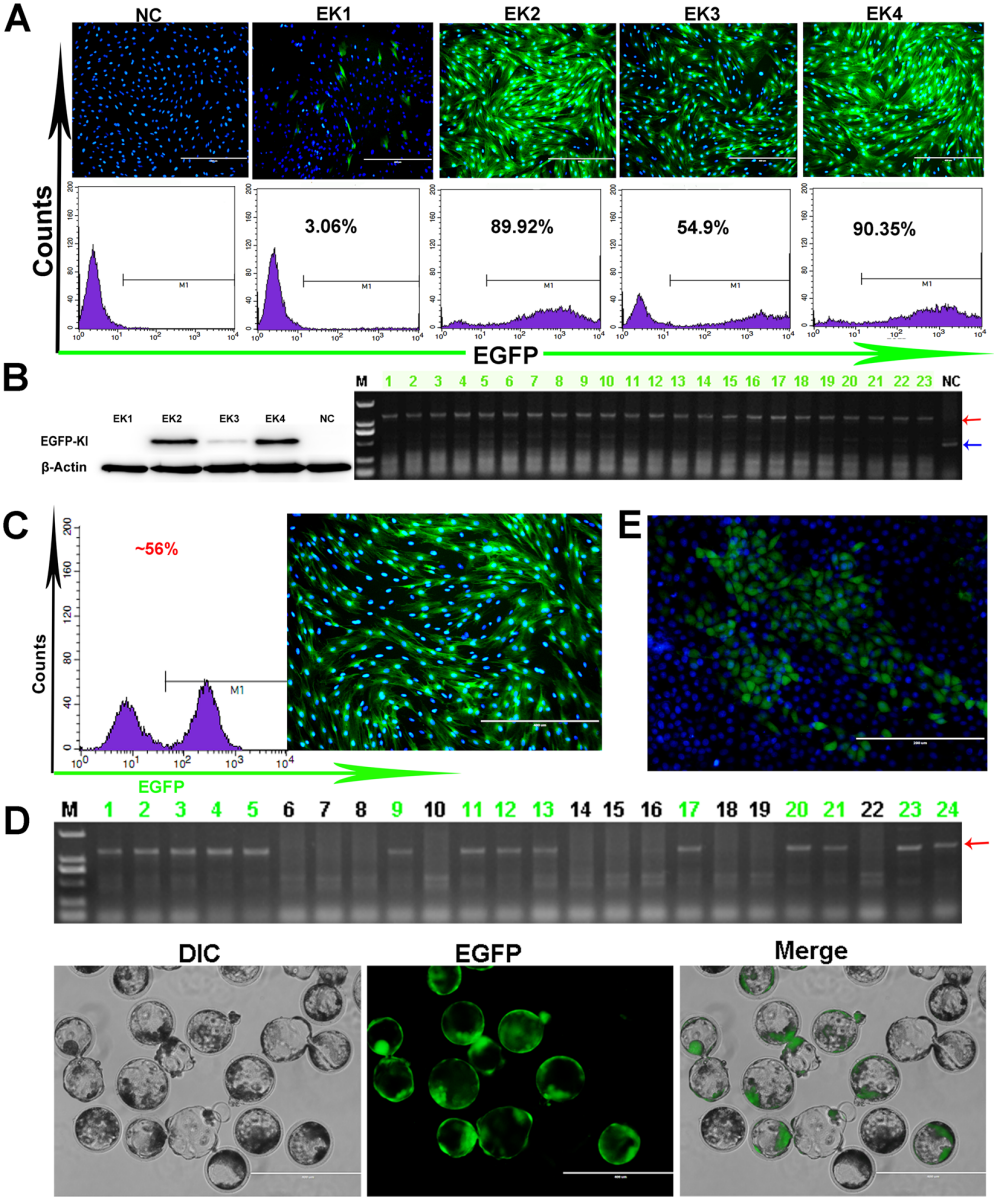
Application of the EGFP-KI reporter PFFs. (**A**) The purity of each EGFP gene site-specific knock-in cell clones was analysed via fluorescence microscopy and confirmed via FACS. (**B**) To confirm of the purity of each cell clone by western blotting (shown on the left); Genomic PCR analysis of the blastocysts (shown on the right); these 23 nuclear donor cells were derived from cell clone-EK4. The results show the high purity of cell clone-EK4. The red arrow indicates the knock-in bands and the blue arrow indicates the wild-type bands. NC: negative control. (**C**) The proportion of mixed nuclear donor cells was analysed via fluorescence microscopy and confirmed via FACS. (**D**) Genomic PCR analysis of the blastocysts (upper). Nuclear donor cells were derived from the mixed nuclear donor cells in fig. C . The red arrow indicates the knock-in bands. After 7 days of culture, the mixed blastocysts were observed by fluorescence microscopy (lower). M: D2000. (**E**) Generation of the EGFP reporter PK-15 cell lines was confirmed via fluorescence microscopy.

As described above, our primary goals involved the selection of genome-modified PFFs to generate transgenic pigs through SCNT. During our screening of the cell clones with the limiting dilution method, we notice that the cell inoculation number had an important effect on the purity and activity of the nuclear donor cell clone that would directly influence the positivity rate, the activity of the embryos and of transgenic pig birth rates. In this study, we found that the purities of the EGFP site-specific knock-in cell clones were strongly affected by the degree of the cell dilution; PFF cell clones with different purities displayed differing apparent EGFP fluorescence intensities via fluorescence microscopy and confirmed by FACS (Fig. 5A). The purities of the cell clones were also confirmed by western blotting (Fig. 5B). The higher-purity EK4 cell clone was used as the donor cell for SCNT, recombinant cells were cultured until blastocyst stage, and 23 blastocysts were harvested for PCR analysis. The results confirmed that all 23 blastocysts were positive for the EGFP knock-in (Fig. 5B).

The visible EGFP site-specific knock-in PFFs can be used to optimize the limiting dilution method. To obtain donor cells with highest purities and activities, different cell numbers (1,500, 2,500, 3,500, 4,500, 5,500, 6,500 and 7,500) were inoculated into 100 mm cell culture dishes. After 9 days, the cell clones in the 100 mm dishes were counted and statistically analysed, and the results showed that the inoculation of 3,500 cells in the 100 mm dish produced the highest purity and activity of the EGFP knock-in positive cell clones (Supplementary Fig. S11).

The EGFP site-specific knock-in PFFs can also be used to accurately evaluate the safety/feasibility of the knock-in site. The EGFP knock-in-positive and wild type (WT) cells were mixed in a specific proportion (e.g., 56%) (Fig. 5C), and the mixed cells were randomly selected for SCNT. After 7 days, the mixed blastocysts were observed by fluorescense microscopy and PCR (Fig. 5D), and the results showed that both the overall blastocyst development rate (12%) and the EGFP-positive blastocyst development rate (11%) were similar to those of the WT group (10%) (Fig. 5D, Table 2).

**Table 2:** The safety evaluation of the gene target site.

Total Nuclear Donor Cell Numbers			Blastocyst Numbers			Blastocyst Development Rate		
EGFP Positive (219 * 56%)	WT (219 * 44%)	Total	EGFP Positive	WT	Total	EGFP Positive	WT	Total
~120	~99	219	14	10	24	12%	10%	11%

## Discussion

Transefection efficiency has always been a key step in the generation of genetically modified large animals, particularly those in which a precise modification is achieved by homology-directed repair. In this study, we improved the electroporation efficiency in PFFs and confirmed that this electroporation system would also obtain high transfection efficiencies in various cell lines. Additionally, we demonstrated that the electroporation system can be used for mRNA and siRNA transfections. These results indicate that electroporation is a versatile transfection system that may be used to explore the biological functions of mRNAs, siRNAs and various cell lines. For example, siRNAs could be designed to suppress several NHEJ key molecules, including DNA ligase IV, KU70, KU80 and XLF^24, 25^, and mRNAs could be designed that increase the expression of key HDR key molecules, such as RAD51 and RAD52^21, 22, 26^. Then, corresponding siRNAs or mRNAs could be introduced into the PFFs with CRISPR/Cas9 and the donor vector by using this transfection system, which may be used to enhance the site-specific knock-in efficiency. Here, we confirmed that the heights and complexities of the multi-peaks in the chromatogram were consistent with the results of the TA cloning experiments and T7E1 assays. This method provides a convenient means of estimating the mutation/cutting efficiency. Several recent studies have shown that highly efficient homologous recombination can occur in pig zygotes through direct zygote injections of Cas9/sgRNA^16, 27, 28^. However, our results showed that the mutation efficiency was only 14% when the Cas9 mRNA and sgRNA were microinjected into parthenogenetic embryos. These results indicated that the injection dose of the sgRNA and Cas9 RNA needed to be optimized further and also highlighted the difference between zygotes and parthenogenetic embryos.

Although the indel efficiency of CRISPR/Cas9 is high, low knock-in efficiency has presented a major bottleneck in the technique’s broad application. There are numerous opportunities and challenges in the field of improving the knock-in efficiencies. Although the positive and negative selection method can be used to improve the knock-in efficiency, the adverse effects of introducing drug selectable marker genes into cells and animals have not been addressed. The possibility that drug selection may harm cells and animals in ways that are currently not understood or that cannot be detected should not be dismissed. Moreover, expression of a drug selectable marker gene in genetically modified cells or species may also interfere with internal gene expression^13, 29^. Selectable marker genes have traditionally been inserted into the genome between loxP sites and removed by using Cre. However, deleting a selectable marker is a laborious process. In this study, we optimized the conditions for a site-specific gene integration at the pRosa26 locus in PFFs with the CRISPR/Cas9 vector and an EGFP targeting donor vector (a non-linearized vector, that does not contain a promoter or selectable marker), for a final efficiency of approximately 29.6%. Furthermore, the promoter-less transgenic method averts several problems, such as unstable phenotypes, unpredictable gene expression and oncogene activation. Our results demonstrate that the CRISPR/Cas9 system can efficiently generate promoter-less and selectable marker-free transgenic PFFs in one step, and it saves time and money whilst favouring promoter-less and selectable marker-free transgenic pigs.

We have also shown that EGFP site-specific knock-in PFFs can be successfully produced by co-electroporation of the Cas9 mRNA, sgRNA and donor vector, thus reducing the cytotoxicity and random integration risk that often accompanies sgRNA/Cas9 DNA-plasmids, because the Cas9-mRNA is easier to be degraded compared with Cas9-plasmids after the gene of interest is targeted/cut. However, the knock-in efficiency was lower than that of the sgRNA/Cas9 DNA-plasmids; this result suggested that an appropriate transfection dose, transfection concentration, timing of transfection and sgRNA-Cas9 mRNA proportion would need to be established to further improve the knock-in efficiency in future investigations.

Previous reports have shown that SCR7, an NHEJ inhibitor and NU7441, a DNA-PKcs inhibitor increase the HDR frequency^2, 19, 30^. However, in our study, the addition of the SCR7 and NU7441 showed no significant effects on the HDR efficiency at the indicated pROSA26 locus in PFFs. The discrepancy between our work and these reports suggest that different species may respond differently to these drugs or alternative NHEJ mechanisms that are not SCR7 orNU7441 sensitive exist in PFFs. Additionally, the size of DNA donors, the timing of drug treatment, the culture environmental conditions and genetic factors may appear to influence the knock-in events and it remains to be tested whether SCR7 has effects when double stranded oligodeoxynucleotide (dsODN) or single stranded oligodexynucleotide (ssODN) is used as the donor. Surely, additional work is needed to confirm the effects of SCR7 and NU7441 in PFFs system because only a single locus was targeted in our present work.

Interestingly, we found that SCR7 significantly promoted PFFs proliferation when the culture concentration was 5 μΜ (Fig. 3E). Additionally, we identified two drugs the cyclin-dependent kinase (CDK) inhibitor, indirubin-3′-monoxime and microtubule polymerization inhibitor, Nocodazole, they could improve the knock-in efficiency at the indicated pROSA26 locus in PFFs. Our results showed that a 2.8-fold increase and a 1.9-fold increase in HDR with Nocodazole and indirubin-3′-monoxime at the indicated pROSA26 locus. These results may indicate that HDR is the major DNA repair mechanism after G2/M phase arrest in PFFs. To further increase the knock-in efficiency, we demonstrate Nocodazole, indirubin-3′-monoxime and RAD51-over expression plasmid as separate tools to efficiently lead to an increase in knock-in efficiency, with highest rates of HDR up to 29.6% in PFFs. Further investigations should be done to optimize the timing of drug treatment, the concentration and combination of these drugs to obtain a higher frequency of homozygous targeting in pROSA26 locus or others, and further refining the process. However, the effects of these drugs treatment on blastocyst development and birth rate of piglets should be performed comprehensively and systematically in subsequent research.

Additionally, after combining different doses of SCR7 and the pEGFP-N1 plasmids (30 μg) into the electroporation system, SCR7 can be quickly introduced into PFFs along with pEGFP-N1 plasmids. We were able to quickly and conveniently evaluate the effect of SCR7 on the electroporation system; we also conveniently assessed SCR7’s cytotoxicity by monitoring numbers and proportions of EGFP-positive cells by fluorescence microscopy.

The off-target mutation is a major concern with the Cas9-mediated gene editing system, thus indicating that CRISPR/Cas9 can tolerate small numbers of mismatches between sgRNA and the target region, particularly when the mismatch is 8–12 bases away from the protospacer adjacent motif (PAM)^31^. Our results indicated that sgRNA91/Cas9 did not induce detectable off-target mutations in the 10 EGFP transgenic PFF cell clones, thus possibly suggesting that the off-target effect of the CRISPR/Cas9 is site-dependent and that CRISPR/Cas9 may be a reliable gene targeting tool for genome modification. Nevertheless, to thoroughly detect a potential off-target effect, whole-genome sequencing analyses or other testing methods should be used for further confirmation. Additionally, DNA nicks^32^, fCas9^33^, truncated gRNA^34^ and RFNs^35^ can be used for gene editing to avoid off-target effects in the future.

Transgenic pigs can be generated by SCNT, but the purity and activity of the nuclear donor cell is key to this process. When the limiting dilution method is used to select the transgenic PFF cell clones, too many inoculated cells lead to a coalescence or cross-linking of the cell clones and reduce the purity of the cell clones. However, too few inoculated cells will weaken the interactions among the cells and decrease their proliferation rate, thereby affecting the quality or activity of the donor cell. The EGFP reporter cell lines enabled us to improve the limiting dilution method and conveniently evaluate the activity and purity of each cell clone through fluorescence microscopy. In this study, we successfully selected the optimal cell inoculation density in 100 mm culture dishes (3,500 cells/dish on average) by using the EGFP reporter PFFs. These cells may provide a reference for other genetically modified animals that are produced by SCNT.

Using the EGFP reporter PFFs, we developed a strategy to evaluate the feasibility and safety of the “safe harbour” locus. We believed that the hybrid transplantation strategy might further exclude human influence; it was more accurate than the separate and individual transplantation method.

Additionally, we introduced sgRNA91/Cas9 into the PK-15 cell line with the EGFP-KI donor and obtained the PK-15-EGFP-reporter cell lines, which stably expressed the EGFP protein (Fig. 5E). It is well known that, a limitation of transgenic PFFs is their lower viability and proliferation rate through serial cell passages. However, cell passaging has a weak influence on the viability and proliferation ability of the PK-15-EGFP-reporter cell line. Compared with cells with transiently co-transfected EGFP plasmids or EGFP random integration cell lines, the PK-15-EGFP-reporter cell line has the follow advantageous features: a precise integration site, a specific copy number, and a higher controllability. With these advantages, the PK-15-EGFP-reporter cell line can be readily used to optimize gene editing tools or to screen recombinants, such as CRISPR/Cpf1^36^, C2c2^9^ and others. The gene editing tools described above can be designed to target EGFP or its expression products, and the factors and conditions that influence these tools can be conveniently analysed via FACS and fluorescence microscopy.

In summary, we confirmed the efficiency and versatility of the transfection system. We also optimized the transfection dosage of the CRISPR/Cas9 and a suitable homology arm length for efficient homologous recombination (HR). With this strategy, we demonstrated that the CRISPR/Cas9 system can efficiently generate promoter-less and selectable marker-free transgenic PFFs in one step, and it saves time and money whilst generating promoter-less and selectable marker-free transgenic pigs. Using the EGFP reporter PFFs, we further optimized the limiting dilution method and established strategies to evaluate the safety and feasibility of the integration site; these analyses established an evaluation system and contributed to the process of generating transgenic pigs. These methods and their parameters can be directly adapted to other mammalian species, and they provide a reference for more sophisticated genome modifications.

## Materials and Methods

### Ethics statement

All animal studies were approved by the Animal Welfare and Research Ethics Committee at Jilin University (Approval ID: 20150315), and all procedures were conducted strictly in accordance with the Guide for the Care and Use of Laboratory Animals. All surgeries were performed under anesthesia, and every effort was made to minimize animal suffering.

#### Plasmid construction

The donor vector contained a 0.5 Kb left homologous arm (HA) and a 1.0 kb right HA (Fig. S5). The HAs were amplified by genomic PCR and cloned into the PUC57 vector. The SA sequence and the EGFP gene were subsequently inserted between the right and left arms.

sgRNAs that targeted the pROSA26 locus were designed using online software, and sgRNA oligonucleotides were annealed and cloned into the PX330 vector (Addgene) using the method described by Zhang at the Broad Institute of MIT.

#### *In vitro* transcription

The T7 promoter was added to sgRNA and Cas9 by PCR amplification. PCR products were used as templates for *in vitro* transcription (IVT) with a MEGAshortscript T7 kit (Life Technologies) and the mMESSAGEmMACHINE T7 ULTRA kit (Life Technologies) according to the manufacturer’s instructions. The sgRNA and Cas9 mRNA were purified using a MEGAclear kit (Life Technologies) according to the manufacturer’s instructions and were eluted in RNase-free water. The concentration and quality of the mRNA were determined by using a NanoDrop 2000 spectrophotometer and by agarose gel electrophoresis.

#### Isolation and culture of PFFs

Twelve 33-day-old fetuses were separated from Large White sows in gestation period, and primary PFFs were isolated from these 33-day-old foetuses of Large White pigs. After removal of the head, tail, limb bones and viscera from the foetal body, the fetuses were cut into little small pieces, digested with a sterile collagenase solution and cultured in Dulbecco’s modified Eagle’s medium (DMEM, GIBCO) supplemented with 10% foetal bovine serum (FBS) at 39 °C and 5% CO_2_ in a humidified incubator.

#### SCNT

The EGFP-KI-positive PFF cells line that were selected by the limiting dilution method were injected into the perivitelline cytoplasms of enucleated oocytes. The reconstructed embryos were activated and cultured to develop into blastocysts. The blastocysts were analysed by fluorescence microscopy (Nikon ts100). High quality blastocysts were collected into a cell lysis solution that contained 0.45% NP_40_ and 0.6% proteinase K, and their genomic DNA was extracted at 56 °C for 1 h and 95 °C for ten minutes in BIO-RAD PCR machine. The lysate was used as the PCR template. To confirm the cell clones and blastocysts for the site-specific knock-in, we assessed the 5′-junction and the 3′-junction by PCR, the primers and sequences are shown in Table S3.

#### T7EI assay

The T7E1 assay was performed as previously described. Briefly, PCR products were purified with a TIANgelMidi purification Kit (TIANGEN, Beijing, China) and were denatured and annealed in NEBuffer 2 (NEB) in a thermo cycler. Hybridized PCR products were digested with T7E1 (NEB, M0302L) for 30 minutes at 37 °Cand subjected to 2% agarose gel electrophoresis.

#### Western blotting

The Cell clones were solubilized in lysis buffer. The protein concentrations were measured with a BCA protein assay kit (Beyotime, Haimen, China); the proteins were separated on 10% SDS-PAGE gels and transferred to nitrocellulose membranes. The membranes were subsequently blocked for 2 h in 5% low-fat milk in PBST. The membranes were incubated with anti-GFP antibody (1:200, Beyotime) for 1 h, washed 3 times with a TBST buffer, and incubated for 1 h with a horseradish peroxidase-conjugated anti-goat secondary antibody (1:1000, Beyotime). After three washes in PBST, the signals on the membranes were acquired with ECL-Plus western blotting reagent.

#### Off-target analysis

Potential off-target sites of the pRosa26 site were detected by PCR and DNA sequencing. The PCR programme was as follows: 94 °C for 5 min; 94 °C for 30 sec; 32cycles of 58 °C for 30 sec and 72 °C for 40 sec; 72 °C for 5 min; storage at 4 °C. The primer sequences are listed in supplementary Table S4. The PCR products that surrounded the off-target sites were purified and digested with T7E1 for 30 min at 37 °C and subjected to 2% agarose gel electrophoresis.

#### Electroporation of PFFs

Approximately 3 × 10^6^ PFFs and 30–60 µg of the corresponding plasmids (25 µg of EGFP-N1, 30 µg of PX330 and 30 µg of PX330 plus 30 µg of donor vector) were suspended in 300 µL of Opti-MEM (Gibco, Grand Island, New York, USA) in 2 mm gap cuvettes, and electroporated by using specified parameters with a BTX-ECM 2001.

#### Selection of PFF cell clones

The cells were inoculated into ten 100 mm dishes at 48 h post-transfection, and the cell inoculation density per 100 mm dishes was 3,500 cells/dish on average. The cell clones were picked and cultured into 24-well plates. After a confluence of 80% or more was reached, 15% of each cell clone was digested and lysed with 10 μl NP_40_ lysis buffer (0.45% NP_40_ plus 0.6% proteinase K) for 1 h at 56 °C and 10 min at 95 °C. The the lysate was used as the PCR template and was subjected to 1% agarose gel electrophoresis. Additionally, the PCR products were sequenced to confirm the knock-in events. The positive cell clones were thawed and cultured in 12-well plates before SCNT.

#### PCR detection

To test cell clones for site-specific insertion of the EGFP gene, we performed a 5′-junction and 3′-junction PCR reaction. These primers are listed in Table S3.

#### Fluorescence detection and flow cytometric analysis

PFFs were electroporated with pEGFP-N1; EGFP-KI-positive cell clones and EGFP-KI-positive embryos were assessed by fluorescence microscopy (Olympus BX51). The harvested cells were washed twice, resuspended in 300 µL of DPBS and analysed using a BD Accuri C6 flow cytometer.

#### Statistical analysis

The data were statistically analysed with GraphPad Prism software (t-test), and a p value < 0.05 was considered statistically significant.

## Electronic supplementary material

Supporting information
