# Supplementary material for: Optimization of a CRISPR/Cas9-mediated Knock-in Strategy at the Porcine Rosa26 Locus in Porcine Foetal Fibroblasts
Source: Sci Rep. 2017 Jun 8;7:3036. doi: 10.1038/s41598-017-02785-y (PMC5465212; doi:10.1038/s41598-017-02785-y)
Supplement: Supplementary file 1 — Supporting information [file 41598_2017_2785_MOESM1_ESM.doc]

# Supporting information

# Optimization of a CRISPR/Cas9-mediated Knock-in Strategy at the Porcine Rosa26­­ Locus in Porcine Foetal Fibroblasts

**Authors:** Zicong Xie^1‡^, Daxin Pang^1‡^, Kankan Wang^1^, Mengjing Li^1^, Nannan Guo^1^, Hongming Yuan^1^, Jianing Li^1^, Xiaodong Zou^1^, Huping Jiao^1^, Hongsheng Ouyang^1^, Zhanjun Li^1^, Xiaochun Tang^1^*

**Affiliations:** 1 Jilin Provincial Key Laboratory of Animal Embryo Engineering, College of Animal Sciences, Jilin University, Changchun, Jilin Province, People’s Republic of China

‡These authors contributed equally to this work.

* Corresponding author: Xiaochun Tang. Email**:** xiaochuntang@jlu.edu.cn

**Address:** 5333#, Xi’an Road, Changchun 130062, China; Tel: (86)431-87835175; Fax: (86) 431-87980131


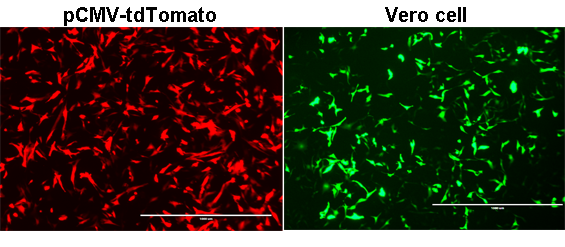


**Figure S1**: Electroporated the pCMV-tdTomato vectors into the PFFs transiently, and the transfection efficiency was analysed by fluorescence microscopy at 24 hours post-transfection (left). Transient electroporation of the pEGFP-N1 vector into the Vero cell lines (right) and EGFP fluorescence was analysed via fluorescence microscopy at 24 hours post-transfection.


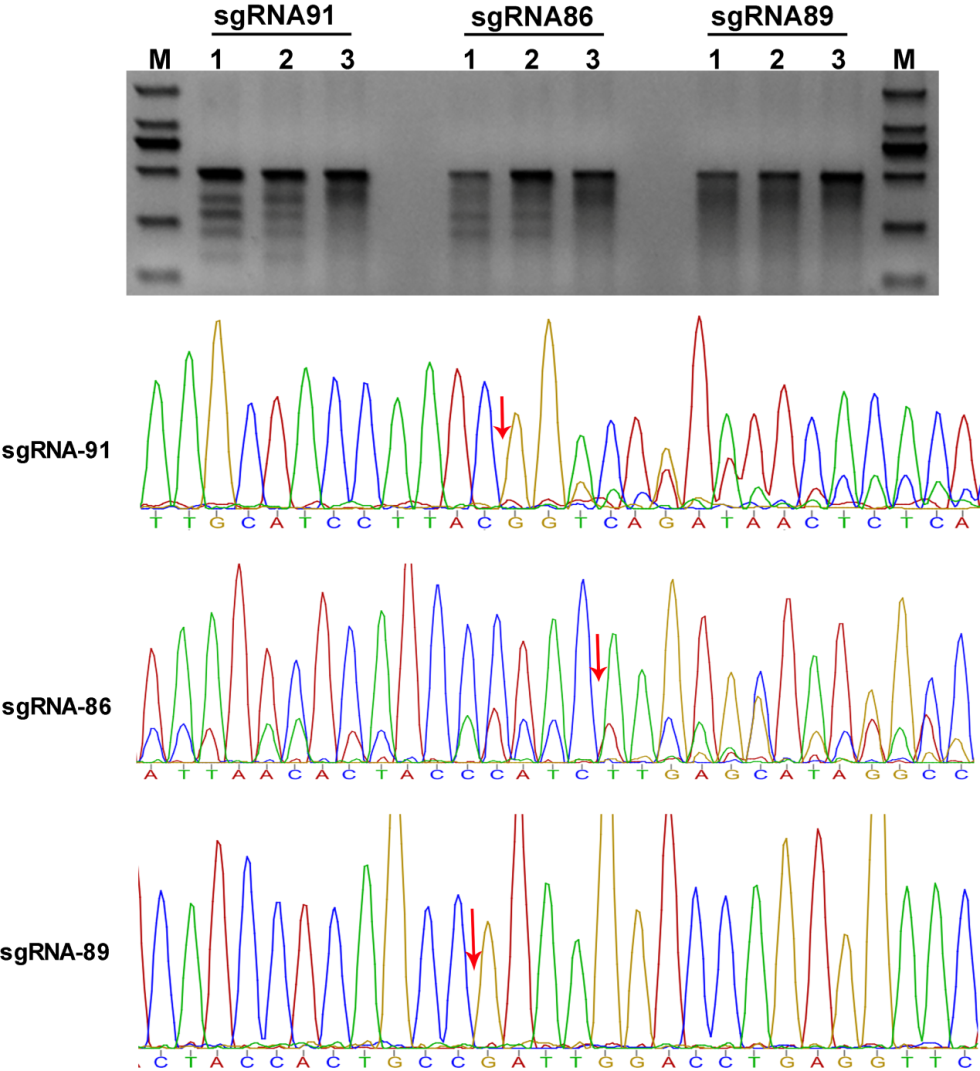

**Figure S2**: The cutting/mutation efficiency of sgRNA86, sgRNA89 and sgRNA91 were evaluated by T7E1 cleavage assay (upper). Sanger sequencing analyses were used to confirm the cutting/mutation efficiency caused by CRISPR/Cas9 (lower). The cleavage sites are labeled with red arrows. M, D2000;.


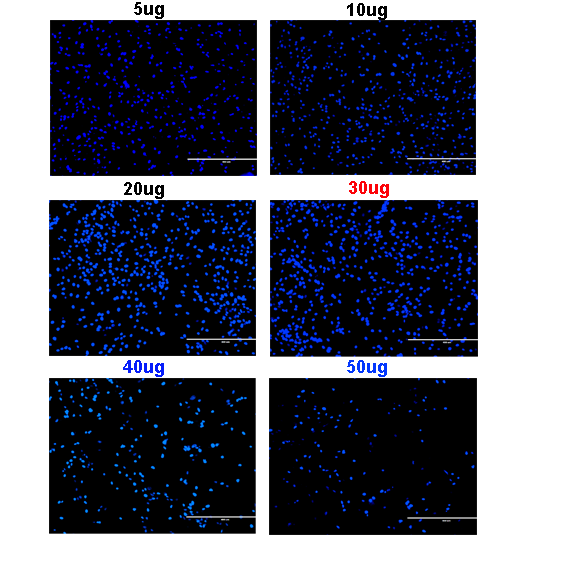


**Figure S3**: Influences of different transfection dosages of the sgRNA91/Cas9 (5 ug, 10 ug, 20 ug, 30 ug, 40 ug and 50 ug) on the cells viability/activity, and nucleus were stained dark blue with Hoechst 33342.


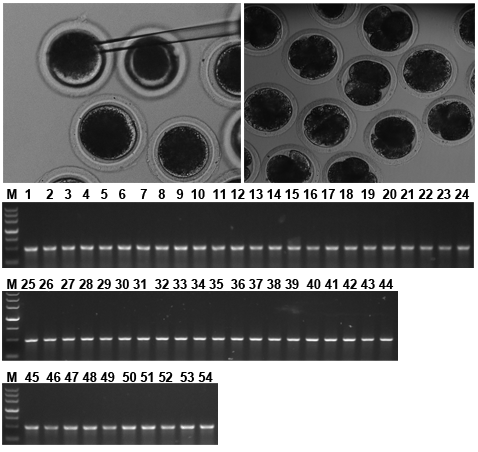


**Figure S4**: The left photograph showed the morphology of the pig oocytes and the microinjection of oocytes, the right photograph showed post-injected oocytes and cultured until blastocyst stage, and in below, electrophoresis result (54 samples) showed that the lysis solution of a single blasocyst mentioned above could be prepared sufficient genomic DNA template for PCR and sequencing, then to evaluate the cutting/mutation efficiency caused by CRISPR/Cas9. M, markerⅢ.


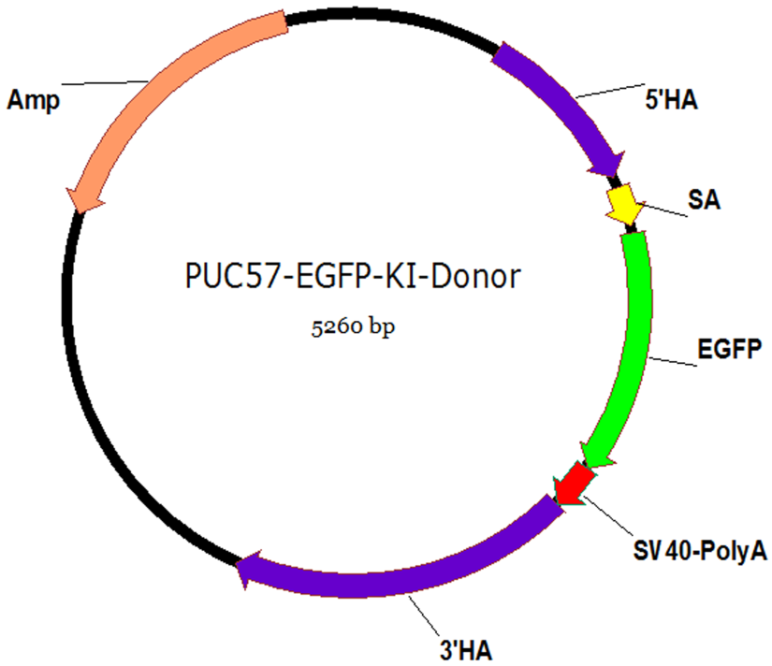


**Figure S5**: The composition and structure of Puc57-pRosa26-EGFP donor vector.


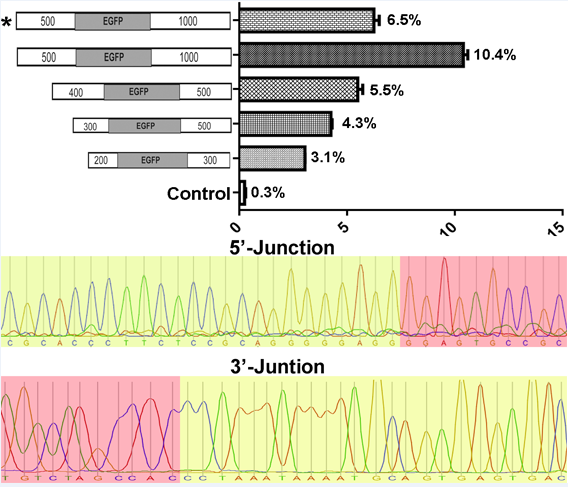


**Figure S6**: knock-in efficiency of these donor vectors with different lengths of homologous arm (upper). * indicate the mRNA-Cas9 transfection group. Sanger sequencing analyses were used to further confirm the EGFP site-specific knock-in events in pROSA26 locus (lower). n = 3. Graphs show the mean ± S.E.M.


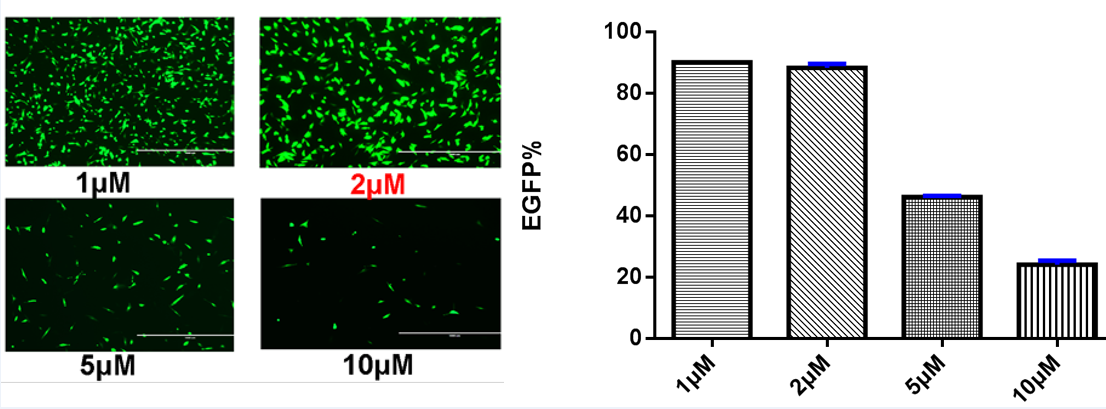


**Figure S7**: The effect of different SCR7 concentrations on the transfection system (fluorescence microscopy) and statistical results of flow analysis (Column chart, n = 3 independent experiments).


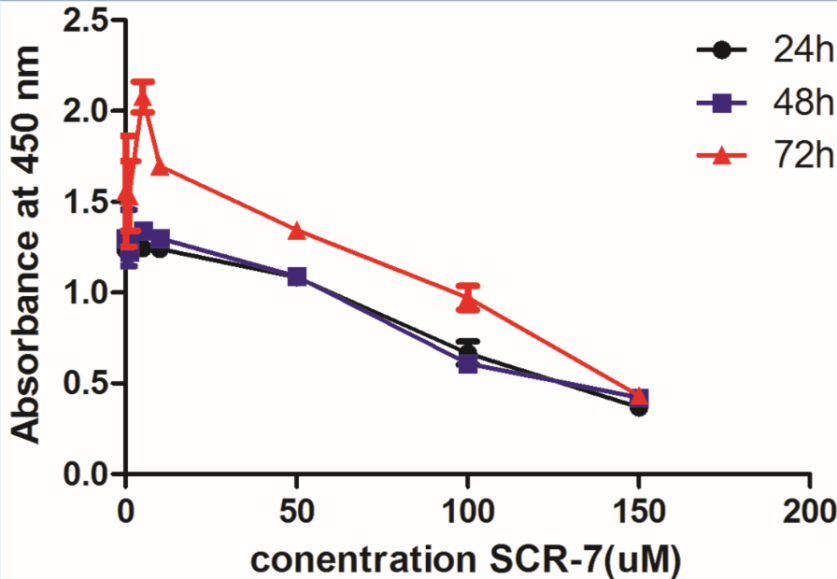


**Figure S8**: CCK-8 analysis to confirm the influence of different concentrations of SCR7 on the PFFs in cell culture system. (line chart. n = 3. Graphs show the mean ± S.E.M.)


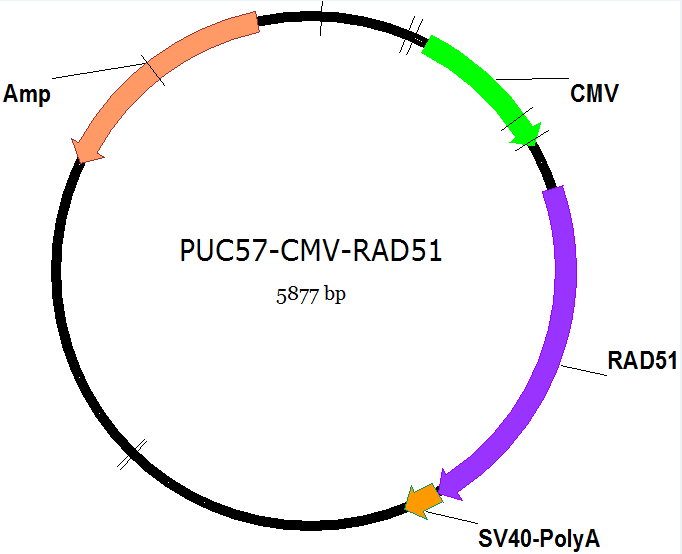


**Figure S9**: The composition and structure of Puc57-pRosa26-EGFP donor vector.


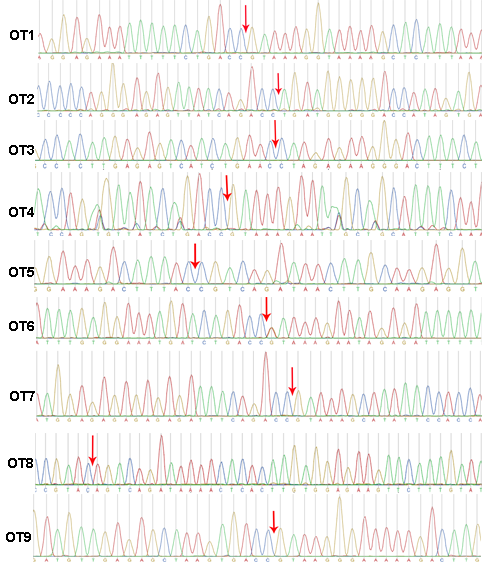


**Figure S10**: Sanger sequencing analyses of PCR amplicons spanning the potential off-target sites were used to further confirm no mutation was detected in these POTS (potential off-target sites). OT1~OT9: 9 potential off-target sites. These red arrows indicate potential target sites.


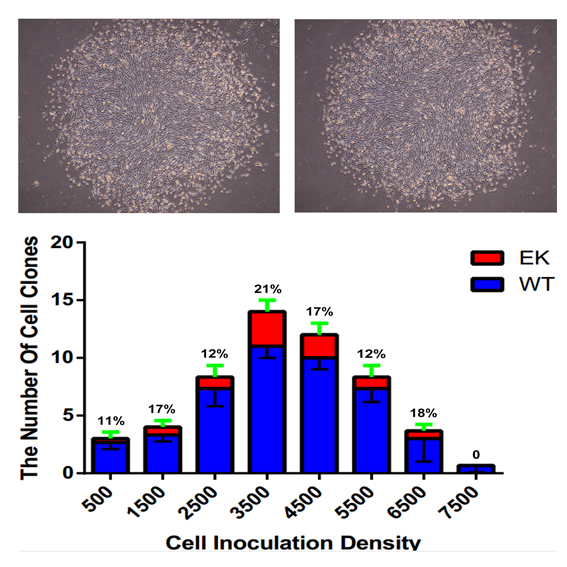


**Figure S11**: Two PFF cell clones (upper). Histogram showed that different cell inoculation numbers (500, 1,500, 2,500, 3,500, 4,500, 5,500, 6,500 and 7,500) could obtain different numbers of cell clone with different purities (lower). n = 3. Graphs show the mean ± S.E.M.


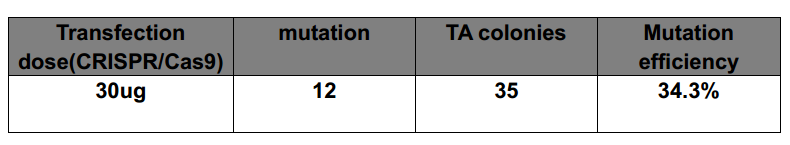


**Table S1**: The PCR amplicons were TA cloned and sequenced to confirm the mutation efficiency caused by sgRNA91 /Cas9.


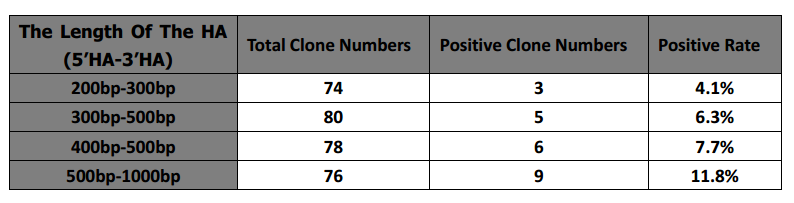


**Table S2**: Statistical results showed that the numbers of EGFP knock-in positive PFFs cell clone generated by different length HA (homologous arm) in 100mm cell dishes.


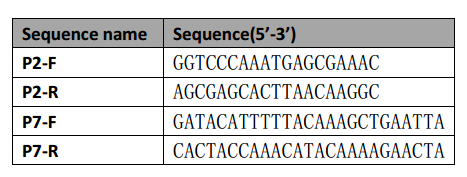


**Table S3**: Knock-in specific primers and Corresponding sequences. Primers-P2 amplified the 5’- junction and primiers-P7 amplified the 3’- junction, respectively.


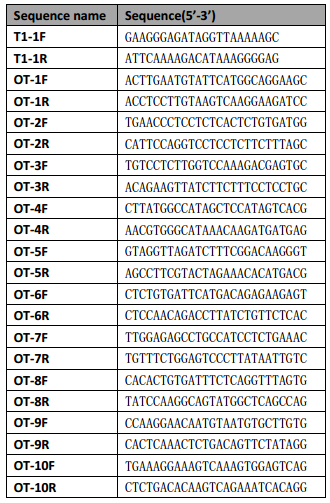


**Table S4**: The primers and sequences were used to analyse the potential off-target sites.
